# Relationship between the Degrees of Itch and Serum Lipocalin-2 Levels in Patients with Psoriasis

**DOI:** 10.1155/2019/8171373

**Published:** 2019-01-21

**Authors:** Norie Aizawa, Yozo Ishiuji, Mitsutoshi Tominaga, Sanae Sakata, Nobuaki Takahashi, Koichi Yanaba, Yoshinori Umezawa, Akihiko Asahina, Utako Kimura, Yasushi Suga, Kenji Takamori, Hidemi Nakagawa

**Affiliations:** ^1^Department of Dermatology, The Jikei University School of Medicine, 3-25-8 Nishishimbashi, Minato-ku, Tokyo 105-8461, Japan; ^2^Institute for Environmental and Gender Specific Medicine, Juntendo University Graduate School of Medicine, 2-1-1 Tomioka, Urayasu, Chiba 279-0021, Japan; ^3^Department of Dermatology, Juntendo University Urayasu Hospital, 2-1-1 Tomioka, Urayasu, Chiba 279-0021, Japan

## Abstract

**Background:**

Lipocalin-2 (LCN2), a protein secreted mainly by activated neutrophils, has been associated with neurodegeneration, obesity, and inflammatory responses. Serum LCN2 concentration has been reported elevated in patients with psoriasis, but lower in patients with atopic dermatitis (AD). Spinal astrocyte-derived LCN2 was found to be involved in enhancement of itch in a mouse model of AD. However, the relationship between LCN2 and itch in patients with psoriasis has not been determined*. Objective*. This study examined the correlation between serum LCN2 levels and the degrees of itch in patients with psoriasis.

**Methods:**

Serum LCN2 concentrations were measured by enzyme-linked immunosorbent assays (ELISA) in patients with psoriasis and AD and in healthy controls. The degree of itch was assessed using a visual analog scale (VAS), and disease severity was determined by measuring psoriasis area and severity index (PASI) and scoring atopic dermatitis (SCORAD). Correlations among serum LCN2 level, VAS, PASI, and SCORAD were analyzed statistically. We further examined the serum LCN levels in psoriasis patients before and after biological treatment.

**Results:**

Serum LCN2 concentrations were significantly higher in patients with psoriasis and AD than those in healthy controls. In patients with psoriasis, serum LCN2 concentrations were significantly correlated with VAS, but not with PASI. In contrast, serum LCN2 concentrations did not correlate with VAS or SCORAD in patients with AD. Serum LCN2 levels in psoriasis patients significantly decreased after the biological treatment along with improvement of VAS.

**Conclusion:**

Serum LCN2 concentration is associated with the degree of itch in patients with psoriasis, suggesting that serum LCN2 may be a useful clinical marker for itch in psoriasis.

## 1. Introduction

Itch is defined as an unpleasant sensation inducing a desire to scratch. Psoriasis is a chronic inflammatory skin disease accompanied by itch in about 60-90% of patients [1–6]. Possible mediators of itch in psoriasis include histamine, opioids, interleukin- (IL-) 31, and brain natriuretic peptide (BNP) [7, 8]. Itch causes distress in patients with psoriasis, not only by impairing quality of life but also by aggravating exanthema due to scratching (Koebner phenomenon) [6, 7, 9]. Despite the importance of controlling itch in patients with psoriasis, effective treatment for itch in psoriasis has not yet been established. Lipocalin-2 (LCN2), also known as 24p3 and neutrophil gelatinase-binding lipocalin (NGAL), is a protein secreted mainly by activated neutrophils [10]. LCN2 has been associated with neurodegeneration, cancer metastasis, insulin resistance, obesity, and inflammatory responses [11–13]. In addition, LCN2 derived from spinal astrocytes has been found to enhance itch in a mouse model of atopic dermatitis (AD) [14]. Serum LCN2 concentration has been reported higher in patients with psoriasis, but lower in patients with AD, than that in healthy controls [15, 16]. LCN2 may contribute to the pathogenesis of psoriasis by modulating neutrophil activities, including neutrophil infiltration [17, 18], migration [19], and activation, inducing neutrophils to release proinflammatory mediators [19]. LCN2 may therefore be a potential target for the treatment of psoriasis [19]. In addition, LCN2 may be involved in the tendency of patients with psoriasis to develop metabolic syndrome [20]. To date, however, the relationship between LCN2 and itch in patients with psoriasis remains unclear. Therefore, the aim of this study was to investigate the correlations among serum LCN2 levels and the degrees of itch and skin inflammation in patients with psoriasis and AD.

## 2. Materials and Methods

### 2.1. Subjects

The study cohort comprised 153 subjects (86 men, 67 women), with mean ± SD age 43.68 ± 15.25 years (range, 18 to 88 years). These subjects were divided into three groups: 59 patients with psoriasis (47 men;12 women; mean ± SD age 52.12 ± 17.73 years); 47 patients with AD (19 men, 28 women; mean ± SD age 40.38 ± 10.51 years), and 47 healthy controls (20 men, 27 women, mean ± SD age 36.38 ± 10.21 years). Patients with psoriasis were diagnosed based on clinical features and/or histopathological findings. Patients with AD were diagnosed as described previously [21].

Psoriasis disease severity was assessed by measuring psoriasis area and severity index score (PASI; 0-72 points) [22], whereas AD disease severity was determined by scoring atopic dermatitis index (SCORAD; 0-103 points) for AD [23]. When PASI ≥ 10 was defined as severe, 40 patients with psoriasis had mild to moderate and 19 had severe disease. When SCORAD ≥ 50 points was defined as severe, 34 patients with AD had mild to moderate and 13 had severe disease. The degree of itch during the previous two weeks was assessed with a visual analog scale (VAS; 0-100%) [24]. VAS was measured at the same day of blood sampling.

Peripheral blood samples were obtained from all study subjects. The blood samples were allowed to clot for 30 minutes before centrifugation for 15 minutes at 1000×*g*. Serum samples were collected and assayed immediately or stored at ≤−20°C.

All subjects provided written informed consent. All procedures were approved by the ethics committees of The Jikei University and Juntendo University Urayasu Hospital. This study was conducted according to the principles of the Declaration of Helsinki.

### 2.2. Enzyme-Linked Immunosorbent Assay (ELISA)

Serum LCN2 concentrations were measured in all study subjects using specific ELISA kits (R&D Systems, Minneapolis, MN, USA), according to the manufacturer's instructions.

### 2.3. Statistical Analysis

All statistical analyses were performed using GraphPad Prism 7 (GraphPad Software Jolla, CA, USA). Data were evaluated by Dunnett's or Bonferroni's multiple comparison tests and Student's *t*-test and correlation coefficients (*r*) by Spearman's rank correlation test. Correlations were deemed weak for *r* from 0.2 to 0.4 and moderate for *r* from above 0.4 to 0.7, with *P* < 0.05 indicating statistical significance.

## 3. Results

### 3.1. Degree of Itch and Serum LCN2 Concentrations in Patients with Psoriasis and AD

Of the 59 patients with psoriasis, 43 had itch while 16 did not (VAS = 0%). Similarly, of the 47 patients with AD, 46 had itch, while only one did not (Table 1).

**Figure 1:**
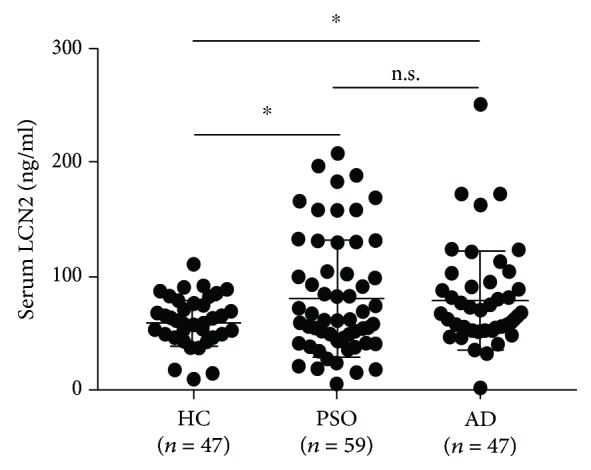
Serum LCN2 levels in patients with psoriasis, patients with atopic dermatitis, and healthy controls. Serum LCN2 levels were significantly higher in patients with psoriasis and atopic dermatitis than in healthy subjects. Bar graphs indicate mean ± SD of serum LCN2 levels. Significant differences were assessed by Dunnett's multiple comparison tests: ^∗^*P* < 0.05.

**Figure 2:**
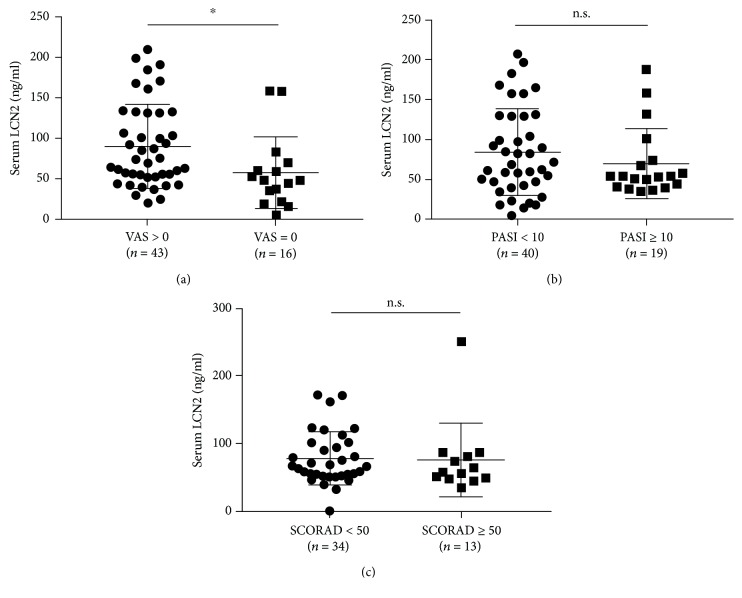
Relationship between serum LCN2 levels and disease severity in patients with (a, b) psoriasis and (c) atopic dermatitis. (a) Serum LCN2 levels were significantly higher in psoriasis patients with than without itch. (b) Serum LCN2 levels did not differ between patients with severe and mild to moderate psoriasis. (c) Serum LCN2 levels did not differ between patients with severe and mild to moderate atopic dermatitis. Bar graphs indicate mean ± SD of serum LCN2 levels. Significant differences were assessed by Student's *t*-tests: ^∗^*P* < 0.05.

ELISA measurements showed that serum LCN2 concentrations were significantly higher in patients with psoriasis (80.08 ± 51.3 ng/ml) and AD (78.32 ± 43.42 ng/ml) than those in healthy controls (59.07 ± 20.18 ng/ml, *P* < 0.05 each), but there was no significant deference in serum LCN2 levels between psoriasis and AD patients (Figure 1). Serum LCN2 levels were also significantly higher in psoriatic patients with than without itch (88.72 ± 51.45 ng/ml vs. 56.86 ± 44.44 ng/ml, *P* < 0.05) (Figure 2(a)), but there was no correlation between serum LCN2 levels and disease severities (PASI or SCORAD) (Figures 2(b) and 2(c)).

### 3.2. Correlations among Serum LCN2 Level, Itch, and Skin Inflammation

**Figure 3:**
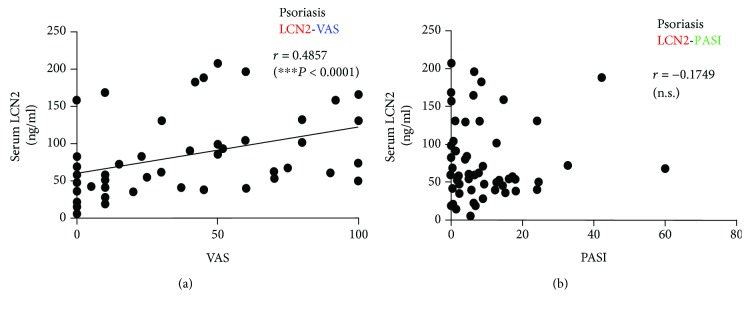
Scatter plots of correlations of serum LCN2 levels with (a) VAS scores and (b) PASI in patients with psoriasis. Serum LCN2 levels in patients with psoriasis were (a) significantly correlated with VAS scores (*r* = 0.4857*P* < 0.0001) but (b) not significantly correlated with PASI. Correlation coefficients (*r*) were determined by Spearman rank correlation tests: ^∗^*P* < 0.05.

**Figure 4:**
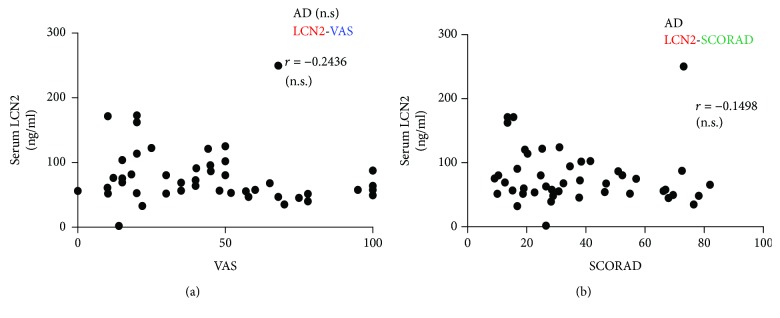
Scatter plots of correlations of serum LCN2 levels with (a) VAS scores and (b) SCORAD in patients with atopic dermatitis. Serum LCN2 levels in patients with atopic dermatitis did not correlate with (a) VAS or (b) SCORAD scores. Correlation coefficients (*r*) were determined by Spearman rank correlation tests.

Serum LCN2 concentrations in patients with psoriasis were significantly correlated with VAS scores (*r* = 0.4857*P* < 0.0001) (Figure 3(a)), but not with PASI (Figure 3(b)). In AD patients, serum LCN2 levels were not correlated with VAS scores or SCORAD (Figures 4(a) and 4(b)).

### 3.3. Comparison of Serum LCN2 Level with Psoriasis Patients between Pre- and Posttreatment

**Figure 5:**
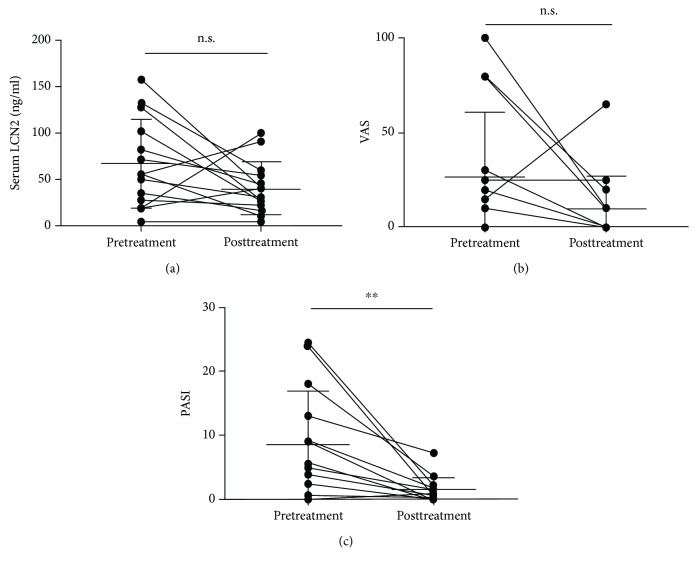
Serum LCN2 levels (a), VAS (b), and PASI (c) in patients with psoriasis pretreatment and posttreatment (*n* = 14). (a) There was a tendency that serum LCN2 levels were lower in psoriasis patients after biologic treatment. However, this trend did not reach statistical significance (*n* = 6). (b) There was a tendency that VAS was lower in psoriasis patients after biologic treatment. However, this trend did not reach statistical significance (*n* = 6). (c) There was a significant difference in PASI with psoriasis between pretreatment and posttreatment. Bar graphs indicate mean ± SD of serum LCN2 levels. Significant differences were assessed by Student's *t*-tests: ^∗^*P* < 0.05 and ^∗∗^*P* < 0.01.

We also compared serum LCN2 levels before and after biologic treatment with 14 psoriasis patients. Four patients were treated with adalimumab, 6 patients with secukinumab, 3 patients with brodalumab, and one with infliximab. There was not a statistical significance but a tendency that serum LCN2 levels and VAS scores were lower in psoriasis patients without itch after biologic treatment (Figures 5(a) and 5(b)). PASI improved significantly after biological treatment (8.493 ± 8.366 points vs. 1.414 ± 1.996 points, *P* < 0.05) (Figure 5(c)).

**Figure 6:**
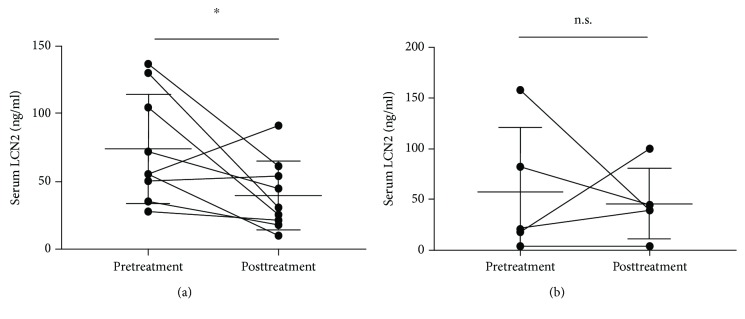
Therapeutic change of serum LCN2 levels between pre- and postbiologic treatment in the patients with itch or without itch. (a) Serum LCN2 levels were significantly lower in psoriasis patients with itch pretreatment than posttreatment (*n* = 9). (b) Serum LCN2 levels did not differ in psoriasis patients without itch between pretreatment and posttreatment (*n* = 6). Bar graphs indicate mean ± SD of serum LCN2 levels. Significant differences were assessed by Student's *t*-tests: ^∗^*P* < 0.05.

**Figure 7:**
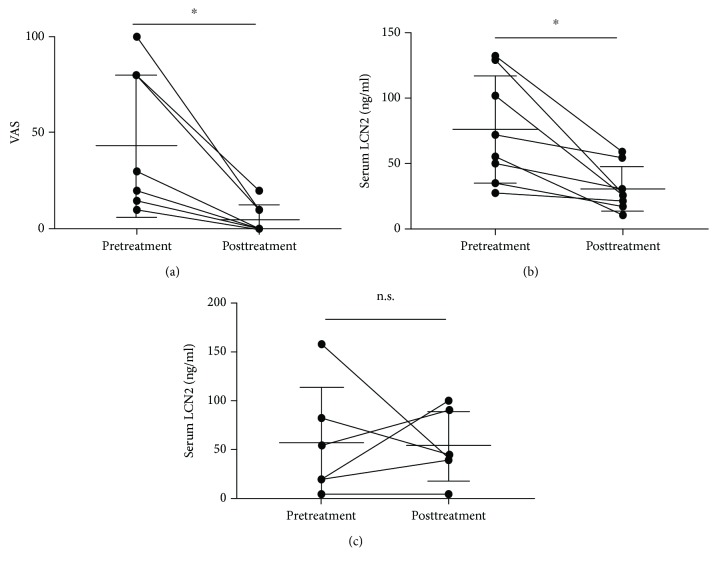
Therapeutic change of VAS and serum LCN2 levels between pre- and postbiologic treatment in the patients who have improvement in itch. (a) VAS was significantly lower in psoriasis patients with itch after treatment than before treatment. (b) Serum LCN2 levels were significantly lower in psoriasis patients with itch after treatment than before treatment (*n* = 8). (c) There was a tendency that serum LCN2 levels were lower in psoriasis patients without itch after biologic treatment. However, this trend did not reach statistical significance (*n* = 6). Bar graphs indicate mean ± SD of serum LCN2 levels. Significant differences were assessed by Student's *t*-tests: ^∗^*P* < 0.05.

Next, we divided the patients into two groups by the presence and absence of itch before biological treatment and compared the change of serum LCN2 levels. Serum LCN2 levels were significantly decreased in psoriasis patients with itch after biological treatment (74.11 ± 40 ng/ml vs. 39.86 ± 25.69 ng/ml, *P* < 0.05) (Figure 6(a)), but there was no significant difference in serum LCN2 levels with psoriasis patients without itch after treatment (Figure 6(b)). Furthermore, to examine whether the serum LCN2 level represents itch sensation, the patients were divided into two groups by the therapeutic effects of itch with the biological treatment. We defined the patients with improved VAS scores as an improved group, while the others with unchanged or increased VAS scores as a nonimproved group. In the improved group, VAS scores and serum LCN2 levels after treatment were significantly decreased than before treatment (43.13 ± 37.12% vs. 5 ± 7.559%, *P* < 0.05) (75.63 ± 40.93 ng/ml vs. 30.81 ± 17.25 ng/ml, *P* < 0.05) (Figures 7(a) and 7(b)). In the nonimproved group, there was no significant change in serum LCN2 levels between before and after treatment (Figure 7(c)).

## 4. Discussion

The present study demonstrated that serum LCN2 levels were higher in patients with psoriasis and AD than those in healthy controls and that only the degree of itch in psoriasis correlated with serum LCN2 levels. LCN2 is an antimicrobial protein [25] mainly secreted by activated neutrophils and associated with neurodegeneration, cancer metastasis, inflammatory responses, insulin resistance, obesity, and atherosclerotic disease [11–13, 26]. Serum LCN2 concentration was shown to be higher in psoriatic patients [15] and LCN2 was found to contribute to the pathogenesis of psoriasis by modulating neutrophil function to enhance T-helper 17-type responses [19, 27]. LCN2 was found to be upregulated in spinal astrocytes in a mouse model of AD, indicating itch sensitization at the spinal level. Although the precise mechanism by which LCN2 induces itch in psoriasis has not been determined, the results of our study suggest that LCN2 enhances itch centrally in psoriasis by a mechanism similar to that in the mouse model of AD.

In addition to its central effect, LCN2 may have peripheral effects, inducing neutrophil infiltration, migration, and activation, all of which have been associated with the pathologic development of psoriasis [19, 27]. Although the relationship between neutrophils and itch has not been fully determined, neutrophil activities have been associated with several pruritogens such as tumor necrosis factor (TNF-*α*) and substance-P (SP). TNF-*α* produced by epidermal keratinocytes is required in the expression of acute and chronic itch in mice [28]. Moreover, SP may be associated with multiple cellular responses, including neutrophil activation [29]. SP has been shown to increase the cutaneous concentration of leukotriene B4 (LTB4) [30], an endogenous mediator of itch in the skin [31]. LCN2 was also found to be overexpressed in the lesional skin of psoriatic patients [19]. LCN2 may be derived from keratinocytes as well as from neutrophils in certain conditions. Epidermal keratinocytes also produce endogenous pruritogens such as TNF-*α*, LTB4, nitric oxide (NO), and thymic stromal lymphopoietin (TSLP) [28, 32–34]. In addition, injection of LCN2 into psoriasiform skin induced by imiquimod increased the levels of mRNA encoding various cytokines and chemokines, including IL-17A, IL-17F, IL-22, IL-12p40, IL-23p19, and CCL20 mRNAs, as well as TNF-*α* and CXCL1 mRNAs [27]. CXCL1 can also induce itch via transient receptor potential vanilloid type 1 (TRPV1) [35]. Taken together, these findings suggest that LCN2 induces the production of pruritogens from neutrophils and epidermal keratinocytes and that LCN2 may be involved indirectly in the pathogenesis of psoriatic itch at the periphery.

Consistent with an earlier study [15], our study found that serum LCN2 levels did not correlate with PASI (*P* = 0.1053). In contrast, another study reported a positive correlation between LCN2 and PASI [20]. These discrepancies may be due to factors such as disease duration and severity and previous medical history and treatment.

We also found that serum LCN2 concentrations were significantly increased in AD patients, despite the lack of correlation between the degree of itch and serum LCN2 level. This finding may be explained by differences in the local and systemic effects of LCN2 in the patients with psoriasis and AD. In contrast to psoriasis, LCN2 expression was not increased in AD skin [36], suggesting that the local effect of LCN2 on itch is limited in AD. In addition, LCN2 has been identified as an adipokine [37]. Serum LCN2 concentrations were found to be increased in patients with metabolic syndrome, with a significant correlation between LCN2 and measures of insulin resistance [37]. Obesity and psoriasis were found to be strongly associated, whereas AD and LCN2 were not [38]. LCN2 may have systemic effects in psoriasis but not in AD. These differences may explain the lack of correlation of serum LCN2 concentration with itch and disease severity in patients with AD.

We examined the change in serum LCN2 levels with psoriasis patients between before and after biologic treatment. The results showed that serum LCN2 levels decreased after treatment in psoriatic patients with itch. In the VAS improved group, there was significantly lower in serum LCN2 levels before than after treatment. Disease severity improved in all patients, but there was no significant difference in the serum LCN2 levels between pre- and posttreatment. Based on our results, we can assume that the decrease of serum LCN2 levels represents the improvement of itch by biologics and serum LCN2 level may be a useful clinical marker for the evaluation of itch in psoriasis patients.

## 5. Conclusion

In conclusion, the present study showed a close relationship between the degree of itch and serum LCN2 level in patients with psoriasis. Serum LCN2 level may represent not only disease severity but also itch intensity. Thus, serum LCN2 level may be a useful clinical marker for itch in patients with psoriasis.

## Figures and Tables

**Table 1:** Demographic and clinical characteristics of patients with psoriasis (PSO), patients with atopic dermatitis (AD), and healthy controls (HC).

	HC	PSO	AD
Age			
Mean	36.38 ± 10.21	52.12 ± 17.73	40.38 ± 10.51
Range	21 to 64	21 to 88	18 to 60
Sex (patient number)			
Male	20	47	19
Female	27	12	28
Itch (patients number)			
With		43	46
Without		16	1
Disease severity (patient number)			
Mild to moderate		40	34
Severe		19	13

## Data Availability

The individual serum LCN2 levels, disease severity, itch intensity, sex, and age data used to support the findings of this study are restricted by the ethics committees of The Jikei University and Juntendo University Urayasu Hospital in order to protect patient privacy. Data are available from Norie Aizawa (Department of Dermatology, The Jikei University School of Medicine, 3-25-8 Nishishimbashi, Minato-ku, Tokyo 105-8461, Japan h19ms-aizawa@jikei.ac.jp), for researchers who meet the criteria for access to confidential data.
